# *Mut9p-LIKE KINASE* Family Members: New Roles of the Plant-Specific Casein Kinase I in Plant Growth and Development

**DOI:** 10.3390/ijms21051562

**Published:** 2020-02-25

**Authors:** Junmei Kang, Zhen Wang

**Affiliations:** Institute of Animal Science, the Chinese Academy of Agricultural Sciences, Beijing 100193, China; kangjunmei@caas.cn

**Keywords:** casein kinase I, phosphorylation, plant specific CK1

## Abstract

Casein kinase I (CK1), a ubiquitous serine/threonine (Ser/Thr) protein kinase in eukaryotes, plays pivotal roles in a wide spectrum of cellular functions including metabolism, cell cycle progression, developmental control and stress response. Plant *CK1* evolves a lineage expansion, resulting in a unique branch of members exclusive to the kingdom. Among them, Arabidopsis Mut9p-LIKE KINASEs (MLKs) target diverse substrates including histones and the key regulatory proteins involving in physiological processes of light signaling, circadian rhythms, phytohormone and plant defense. Deregulation of the kinase activity by mutating the enzyme or the phosphorylation sites of substrates causes developmental disorders and susceptibility to adverse environmental conditions. Recent findings suggest that MLKs have evolved as a general kinase that modifies transcription factors or primary regulatory proteins in a dynamic way. Here, we summarize the current knowledge of the roles of *MLKs* and *MLK* orthologs in several commercially important crops.

## 1. Introduction 

Casein kinase I (CK1), a highly conserved serine/threonine (Ser/Thr) protein kinase, has been identified ubiquitously in eukaryotes ranging from yeast to human since the 1970s [1,2,3,4]. The dual-specificity Ser/Thr kinases evolutionary conserved on both structure and function in eukaryotes have been identified as a family of monomeric kinases [5,6,7,8,9]. In mammals, seven CK1 isoforms (alpha, beta, gamma1-3, delta and epsilon), together with the various splice variants, are involved in a variety of cellular processes, such as chromosome segregation and cellular differentiation, by phosphorylating a wide range of key regulatory proteins [9,10,11,12,13]. 

Plant CK1 evolved an ancient lineage duplication event, resulting in the division of CK1 into two subsets, i.e., casein kinase 1 like (CKL) and a unique group with members exclusively from plant species [14]. The latter subset has attracted great attention from plant scientists in the last decade, and functional characterization of several members from model plants and crops has revealed their essentiality for plant growth and development. Among them, Arabidopsis Mut9p-LIKE KINASEs (MLKs) appear to act as a general kinase to orchestrate a wide range of developmental and stress response pathways, such as light signaling, circadian rhythms and phytohormone [3,4,15,16,17,18]. 

The purpose of this review is to provide an overview of the roles of plant *CK1*, especially the *MLK* family members in growth and development. We summarize the regulation of *CK1* expression and the kinase activity, and the phosphorylation target sites, as far as the available literature permits. In addition, multiple CK1-modulated pathways are discussed with a focus on light signaling, phytohormone, circadian clock and stress response based on the recent research achievements of *CK1* in model plants Arabidopsis and rice. 

## 2. Plant-Specific *CK1* in Arabidopsis and Major Crops

Plant genomes as compared with other eukaryotic organisms encode a large number of CK1 protein kinases [19]. For example, Arabidopsis and rice, model dicot and monocot, have 17 and 15 CK1 encoding genes, respectively [20,21]. These members are grouped phylogenetically into two main clusters, i.e., *CK1-like* (*CKL*) cluster and a plant-specific *CK1* cluster containing members exclusively from plants [14,21]. For the latter cluster, the first characterized member was *Mut9p* in the green alga *Chlamydomonas reinhardtii* [14]. Four Arabidopsis (*MLK1-4*) and six rice *CK1s* belong to the cluster [21]. The *MLK* homologs in angiosperm including amborellales, chlorophyta, lycopodiophyta, monocot and dicot showed a tendency to increase from lower to higher plants (Table 1), suggesting an expansion during evolution probably due to the sessile life. This review described mainly the recent findings of *MLKs* and *MLK* orthologs in rice and several crops. 

**Figure 1:**
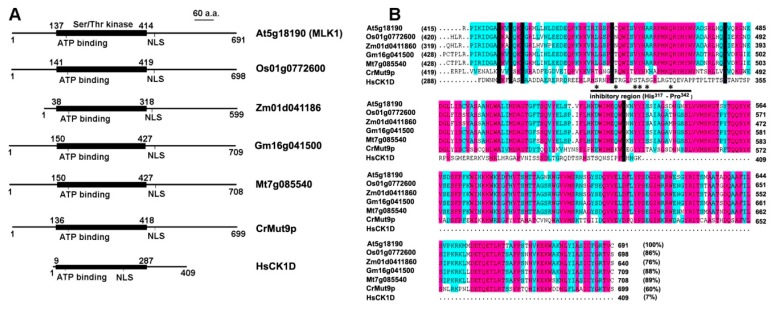
Functional domains and the C-terminal sequence alignment of MLK homologs with human CK1 delta (CK1D). (**A**) Functional domains of CK1 in plants and human. The CK1 family members share a highly conserved (63% to 92% identity) catalytic domain (Ser/Thr kinase domain) of about 280 amino acids. In addition to the kinase domain, the representative kinases contain an ATP-binding site and a nuclear localization signal (NLS); (**B**) Alignment of the C-terminal sequence (the non-catalytic region). The sequence identity of the C-terminal segment to MLK1 (At5g18190) is bracketed. The inhibitory region of human CK1D is underlined (from histone 317 to proline 342), and the potential phosphorylation sites are indicated with star. At, *Arabidopsis thaliana;* Cr, *Chlamydomonas reinhardtii*; Gm, *Glycine max*; Hs, *Homo sapiens*; Mt, *Medicago truncatula*; Os, *Oryza sativa*; Zm, *Zea mays*.

Phylogenetically apart from CKL protein kinases, the MLK homologs in higher plants are evolutionarily conserved (>60% identity) and the region of homology is largely limited to the catalytic motif containing the canonical domains of eukaryotic protein kinases [14]. The Ser/Thr kinase domain contains putative kinase catalytic loop, substrate recognition region and ATP-binding site [22,23], which is supportive of the finding that the plant CK1 uses ATP as phosphate donor to phosphorylate substrate proteins [24]. Interestingly, the N-terminal extension with varying length (from 20 amino acids to up to 410 amino acids), which is absent in animal CKI isomers, differs from one another [14,16] (Figure 1A), whereas the carboxyl (C)-terminal non-catalytic region (about 280 amino acids) is highly conserved (Figure 1B). In contrast, mammalian CKI family members possess a highly conserved amino (N)-terminal domain, but a variable C-terminal extension [23]. Both MLK homologs and human CKI are positively charged, suggesting that these kinases prefer acidic substrates such as serine and threonine residues.

## 3. Regulation of CK1s Expression and Activity 

Although MLKs split from CKLs [21,25], they appear to share similar catalytic structure and mechanism. Similar to animal CK1 enzymes which have been described as constitutively active [24], the spatial and temporal expression pattern of *MLKs* and rice homologs showed that these genes were ubiquitously expressed in all tissues at different developmental stages (http://bbc.botany.utoronto.ca/efp) [3,26]. The catalytic activity of CK1 relies on the conserved amino acid residues which are critical for the three-dimensional structure. The kinase domain of MLK homologs is about 60% identical to that of CKL and the predicted three-dimensional structure resembles the typical CK1 [16]. The structural model of Mut9p uncovered a group of residues determining the Mut9p specificity in association with the histone H3 tail. Among them, Asp 266 (D266) at the position equivalent to the substrate-binding pocket of CK1 was associated with phosphorylatable histone residue T3 [14]. Mutation of the conserved Asp, D267 in the case of MLK4/ PPK1 (photoregulatory protein kinases), caused the elimination of *in vitro* catalytic activity of MLK4/PPK1 [16]. Moreover, an invariant Lys residue (K174 of Mut9p or K175 of MLK4/PPK1), which is implicated in anchoring and orienting the ATP phosphate donor [27], was indispensable for *in vitro* kinase activity in the green alga and Arabidopsis, respectively [14,16]. It is likely that the abolishment of kinase activity by replacing the conserved amino acids is attributed to the alteration of the enzyme structure or substrate specificity determination.

CK1 activity is modulated by inhibitors and the effectors affecting the kinase localization and compartmentalization. Several ATP-competitive small molecules have been identified and characterized as CK1-specific inhibitors in animals [28]. Among them, CK1-7 (N-2-aminoethyl-5-chloroisoquinoline-8-sulfonamide) is the first ATP-competitive inhibitor with no selectivity towards CK1 isoforms [29]. Application of CKI-7 has effectively eliminated the catalytic activity of CK1 in a wide spectrum of plant species, such as rice, broccoli and *Chlamydomonas* [14,30,31]. PF670462, a highly CK1-selective inhibitor, and small molecule IC261 have been applied in investigation of CKL-mediated circadian rhythm [7,32]. Recent chemical screening has demonstrated that PHA767491, an animal CDC7 (cell division control protein 7) inhibitor, and analogs, such as AMI-23, -212 and -331, inhibited CK1 activity [20,33]. Interestingly, mammalian CK1 activity was also affected by inhibitory auto-phosphorylation occurring especially in the highly divergent C-terminal domain and occasionally in the kinase domain. C-terminus truncation elevated kinase activity [34], suggesting the inhibitory effect of auto-phosphorylation was overcome. In spite of a long C-terminus with more potential phosphosites than the non-plant species, MLKs lack the inhibitory auto-phosphorylation domain [16], implying that plants have a distinct or more complicated self-regulation mechanism. Regardless of the report that the nuclear plant kinases were also localized in the cytosol [35], Mut9p, MLK1-4 and EL1 (early flowering 1) have been experimentally shown to reside exclusively in the nucleus [3,16,22,36,37]. These findings suggest that MLK homologs modulate phosphorylation-dependent regulations predominantly in the nucleus, where kinase and substrate are in close proximity.

## 4. Target Proteins Phosphorylated by MLK Homologs

Considerable efforts have been made to uncover plant phosphoproteomes, but the phosphorylation events provided are far from complete due to the fact that phosphoproteomic approaches including *in vivo* and *in vitro* methods are limited in precisely localizing the phosphorylation sites on the proteins. Several databases such as PhosPhAt [38] and P3DB [39] provide information of mass spectrometry-based phosphorylation sites identified or predicted from several plant species. Currently, 56, 857 phosphorylation sites accounting for only 2.6% of the predicted ones including serine, threonine and tyrosine sites have been experimentally determined in Arabidopsis using PhosPhAt 4.0 [40] (http://phosphat.uni-hohenheim.de/statistics.html). Further verification of these phosphorylation sites would be beneficial for the functional characterization of the phosphorylation events. With the rapid development of methodology for protein phosphorylation site(s) identification, a new generation MS instrument, such as quadrupole Orbitrap high-resolution Mass spectrometry, has been used to target hundreds of peptides within one single LC-MS/MS experiment and generated full MS/MS spectra containing sufficient fragmentations for precise localization of the phosphorylated residues [16,41]. 

As plant *CK1* is ubiquitously expressed in all tissues and the protein is localized in the nucleus and/or the cytoplasm, it is not surprising that a broad range of proteins including histones and signaling component proteins have been identified as CK1 targets in model plants and crops. Table 2 enlisted an exhaustive list of CK1 substrates identified in the angiosperm during the last decade. 

### 4.1. Histones Targeted by MLK Famliy Members

Histones are the main protein components of chromatin, a tightly packed higher order structure in the eukaryote nucleus. The basic structure unit of chromatin is the nucleosome that is composed of 147 bp DNA wrapped around an octamer consisting of two copies of each of the four core histones (H3, H4, H2A and H2B) linked by histone H1 [42]. In addition to the globular domain, each of the core histones has a tail protruding from the nucleosome and most of the post-translational modifications including phosphorylation occur at these amino-terminal tails (reviewed by [43,44,45]). As a reversible process, phosphorylation is one of the most important ways of modulating a chromatin structure and accessibility by regulating the switches between condensed and relaxed states of the chromatin higher order structure (reviewed by [46,47,48]). 

Identification of upstream kinases and the target site(s) of individual enzyme is complicated due to the fact that there are numerous candidate kinases responsible for histone phosphorylation and that multiple sites of plant histones are potential phosphorylation targets. In plants, histone phosphorylation modulated by the canonical kinases, such as AtAurora3 and AtHaspin, has been recently reviewed [43,46,49]. *In vitro* assay showed that *Chlamydomonus* Mut9p and Arabidopsis MLK1 and MLK4 phosphorylated histones including H3, H2A and H4, albeit at different intensity [14,22,37]. Partial depletion of phosphorylated H3 at T3 (H3T3ph) was observed in both *mut9* mutant strain and Arabidopsis *mlk1mlk2* double mutant [14,22], confirming that Mut9p and MLK1/MLK2 are responsible for *in vivo* H3T3 phosphorylation in the green alga and Arabidopsis, respectively. The existence of other kinases, especially the paralogos, may explain the incomplete loss of H3T3ph in the mutants. Additionally, MLK4 was reported to phosphorylate H2AS95 residue *in vitro*, while the global level of phosphorylated H2AS95 did not alter clearly in the loss-of-function *mlk4* mutant [37], implying that H2AS95 is not likely the main target of MLK4 in Arabidopsis. Collectively, histones are phosphorylated by MLK homologos in model plants *Chlamydomonus* and Arabidopsis. 

### 4.2. Signaling Components Targeted by Plant CK1 

In addition to the conserved histone residues, plant CK1 including CKL and MLK family members have been predicted, based on structural analysis, to have a broad range of substrates. For example, a rice lipase and an integral negative regulatory domain (NRD) of dehydration-responsive element-binding protein 2A (DREB2A) in Arabidopsis were potential phosphorylation targets of CKL protein kinases [50,51]. Arabidopsis CKL3/CK1.3 and CKL4/CK1.4 phosphorylated photoreceptor CRY2 (CRYPTOCHROME 2) at S587 and T603 *in vitro* in blue-light dependent manner [21], and CKL2 physically interacted with and phosphorylated ADF4 (actin depolymerizing factor 4) in the regulation of actin filament reorganization and stomatal closure [52]. Recently, CKL4 was found to regulate circadian clock by phosphorylating the transcriptional repressor PRR5 (PSEUDO-RESPONSE REGULATIOR 5) and TOC1 (TIMING OF CAB EXPRESSION 1) in Arabidopsis [20]. In sesame and cotton, CKL protein was involved in phosphorylation of SebHLH (the basic region/helix-loop-helix transcriptional factor) [17] and GhTCP15 (TEOSINTE BRANCHED1/CYCLOIDEA/PCF 15) [53], respectively. In the last decade, extensive studies have focused on the functions and regulatory mechanisms of MLK family members in model plant Arabidopsis and rice, and a wide range of substrates have been identified primarily as transcription factors or regulatory proteins involving in basic physiological processes (Table 2). 

## 5. Biological Functions of Plant CK1

Phosphorylation of an amino acid residue can have multiple consequences, including change of enzyme activity, subcellular localization, or protein stability, which affect the interaction with other molecules (protein, DNA, RNA, etc.) and eventually alter the biological functions. In non-plant eukaryotes, the CK1-dependent phosphorylation has been shown to play diverse biological roles [9,11], but the research of plant CK1 lags far behind. The main reason is that compared with animals, plants have a larger number of CK1 encoding genes, which share a high degree of redundancy in both the catalytic and regulatory domains relative to the animal systems. A steadily growing list of both CK1 substrates and the interacting partners adds another level of complexity in dissecting the kinase functions in plants. 

An increasing number of proteins involving in plant growth and stress response have been confirmed as MLK targets in Arabidopsis via mutating the kinase activity site or the phosphosites of the target proteins (reviewed in [57,58,59]). These findings suggest that *MLKs* act as a general kinase in a myriad of cellular processes of plant growth and development. Indeed, *mlk* quadruple mutant is lethal [16,60], supporting the vital nature of *MLKs* in Arabidopsis. The role of the canonical plant CK1 in phosphorylation-dependent regulation of chromatin and cell cycle has been reviewed [43,46,49]. We described, here, the recent findings on the functions of plant *CK1,* especially *MLK* family members in light signaling, circadian rhythm, phytohormone and stress response. The information provides a connection among the molecular events mediated by plant CK1 through direct/indirect modification or interaction with the target proteins. 

### 5.1. Light Signaling 

Plants perceive and response to light signal, a major developmental cue, using multiple families of photoreceptors including phytochromes and cryptochromes, which absorb red/far-red light and blue light, respectively. It has been documented that the stability of CRY2 was negatively regulated by CKL-mediated phosphorylation [21,61] and light signaling components, including HY5 (long hypocotyle 5), HFR1 (long hypocotyl in far-red 1), COP1 (CONSTITUTIVE PHOTOMORPHOGENIC 1) and PIF1 (PHYTOCHROME-INTERACTING FACTOR 1) were phosphorylation targets of CK2 [5], suggesting that phosphorylation plays critical roles in modulating the photoreceptors as light sensors. Recently, two independent studies have revealed the delicate coaction of phytochrome-cryptochrome by PPK/MLK-mediated phosphorylation of PIF3 and CYR2 [58]. Compared with *ppk124* triple mutants, which showed a reduced level of light-induced PIF3 phosphorylation and degradation, more robust reduction was detected in *PPKs* quadruple-knockdown transgenic lines [15], suggesting that *PPKs/MLKs* in Arabidopsis collectively promote light-induced degradation of PIF3 by phosphorylation. The triple mutants of *ppk123/124/134* also displayed hypersensitivity to red light, which was strongly suppressed by mutating *phyB*, suggesting the hypersensitivity is attributed to the reduced degradation of phyB [15]. In an accompanying paper, the level of phosphorylated blue-light receptor CRY2 was clearly reduced in the *ppk* triple mutants with photo-hypersensitivity to blue light. PPKs were also shown to interact with and catalyze the blue light-dependent phosphorylation of CRY2, although individual kinase preferred different phosphosties. Substitution of several Ser/Thr residues at the phosphosites of CRY2 resulted in abolished phosphorylation [16]. Thus, PPKs/MLKs are the major kinases catalyzing the phosphorylation of the two types of photoreceptors in Arabidopsis. Notably, the binding affinity of PPK1 with PIF3 was not affected by deletion of the C-terminal domain of PPK1 [15]. However, the C-terminal of PPKs is indispensable for the interaction with CYR2 in response to blue light [16]. It is tempting to speculate that the C-terminal domain of PPKs is likely to contribute to the receptor discrimination of blue-light from red-light as light spectrum shifts. Expressing phosphor-deficient and phosphor-mimic forms of the substrates in the corresponding mutant backgrounds could help to elucidate the function of CK1-mediated phosphorylation in light signaling. 

### 5.2. Circadian Clock

Like most organisms, plants have evolved an internal timing mechanism, the circadian clock, which is an autoregulatory, endogenous biological cycle with a period of approximately 24 h. A functional and accurate clock-dependent synchronization of physiology with the environment allows for efficient allocation of resources and optimum fitness. CK1 has been known to affect timekeeping across metazoans and fungi [9,62]. Early evidence of the implication of plant *CK1* in circadian clock was found in a unicellular marine algal species *Ostreococcus tauri* [7]. The basal alga of the green lineage contained only one *CK1* with diurnal fluctuation. Overexpression *O. tauri CK1* caused a long-period phenotype and treatment with chemical inhibitor altered circadian period [7]. 

In higher plants, the clock output processes, such as flowering time and hypocotyl elongation, are clearly affected in *CK1*-deficient mutants. For example, mutation of rice *EL1/Hd16* (heading date 16) caused earlier flowering [3,4]. Hd16 was found to phosphorylate OsPRR37 (*Oryza sativa* pseudo-response regulator 37), an ortholog of Arabidopsis circadian clock components PRRs, and act upstream of flowering regulators *Ghd7* (*Grain number, plant height, and heading date 7*) and *Hd2* [55]. In contrast, Arabidopsis *mlk4* and the higher order of *mlk4-* combined mutant(s) flowered late with retarded elongation of hypocotyl [60,63]. The proteome-wide analysis revealed physical interaction between the rhythmic oscillators and protein kinases in Arabidopsis [60]. Among them, MLKs were shown to interplay with the evening complex components ELF3 (EARLY FLOWERING 3) and ELF4 in the assay of affinity purification and mass spectrometry (AP-MS) [60]. MLK1 and MLK2 regulated hypocotyl elongation by interacting with CCA1 (CIRCADIAN CLOCK ASSOCIATED1), which bound to the promoter of *DWF4* (*DWARF4*) [63]. MLK4 interplayed with CCA1 to affect the expression of *GIGANTEA* (*GI*), a positive flowering regulator [37]. Moreover, CKL4 phosphorylated the clock regulators, PRR5 and TOC1, and the circadian period of Arabidopsis was lengthened by simultaneous knockdown *CKLs* [20]. In *prr5 toc1* mutants the period-lengthening effect was attenuated by inhibition of CK1 [64].

Therefore, plant CK1 plays a critical role in regulating the circadian clock via posttranslational modification of the central clock regulators CCA1 and PRRs (including TOC1), as it does in animal systems. Future studies aiming to decipher the interacting network between the circadian components targeted by plant CK1 should broaden our knowledge of the kinase in the epigenetic regulation of circadian rhythm.

### 5.3. Phytohormone

Phytohormones are generally small organic molecules that regulate both the physiological and developmental processes of plants through a complex network of signal transduction pathways. This section described the recent findings on the CK1-mediated phosphorylation of signal transduction components and the downstream proteins of phytohormone gibberellin (GA), auxin and abscisic acid (ABA). 

An increasing body of experimental findings has demonstrated the involvement of *CK1* in GA signaling pathway. Mutation of rice *EL1* caused enhanced GA response with taller plant height. EL1 phosphorylated rice DELLA protein SLR1 (SLENDER RICE 1) and mutation at its phosphorylation site altered GA signaling [3]. In contrast, Arabidopsis *mlk1mlk2* double mutant with shorter hypocotyl was hyposensitive to GA. Although the phosphorylation target(s) of MLK1/MLK2 are not known, both kinases were found to interact with RGA, a DELLA protein REPRESSOR OF ga1-3, to regulate hypocotyl elongation [63]. These observations suggest that *CK1* plays a critical role in regulating the activity or turnover of GA-responsive transcription factors. 

*CK1* has been implicated to regulate auxin synthesis or signaling pathways in plants. For example, rice *OsCKI1* knockdown mutants displayed abnormal root development with altered auxin content [31]. Comparison of two rice cultivars, Asominori and NIL (*ltg1*), representing dominant *LTG1* (*Low Temperature Growth 1*) allele with functional *CKL* and the *ltg1* allele, respectively, revealed that the latter possessed higher content of IAA (indole-3-acetic acid) than the former. Consistently, the shoot growth of NIL (*ltg1*) was less sensitive to NAA (1-naphthylacetic acid) treatment and was less affected by the auxin transport inhibitor NPA (N-1-naphthylphthalamic acid) relative to Asominori [65], suggesting that rice CKL encoding gene *LTG1* affects plant growth through an auxin-dependent process. Research on cotton *CKL* homolog showed that compared with Col-0, the IAA content in *GhCK1* overexpressing Arabidopsis explants was higher, while lower in *GhCKI* RNAi line, suggesting a correlation between *GhCK1* expression level and IAA content [18]. GhTCP15 was identified as the substrate of GhCK1, and its phosphorylation affected IAA content by modulating the transcription of *GhPIF4* [53]. Further identification and functional characterization of CK1 substrates relevant to auxin synthesis or transport should help in understanding the mechanism of CK1-mediated phosphorylation in auxin signaling. 

ABA is an important phytohormone in plant response to adverse environmental conditions by regulating stomata opening and closure. CK1-mediated phosphorylation of the transcriptional factors involving in ABA signaling appears to play a role in plant stress response or adaption. Early research found that application of ABA induced *SeCK1* transcription in developing sesame seeds [17]. Deletion of Arabidopsis *CKL2* caused stomatal closure less sensitive to ABA and phosphorylation of ADF4 (actin depolymerizing factor 4) by CKL2 inhibited actin filament disassembly [52]. Recently, MLKs/AELs (Arabidopsis EL1-like) have been reported to phosphorylate ABA recepetors PYR/PYLs (PYRABACTIN RESISTANCE/PYR1-LIKE). Deduced phosphorylation level of the receptors mimicked the hypersensitivity of *mlk* triple mutants to ABA treatment [2], indicating that MLK-mediated phosphorylation negatively regulates ABA response. Thus, the altered sensitivity of the *CK1*-defective mutants to ABA is supportive of the implication of *CK1* in ABA signal transduction although how these ABA-involving processes are connected has yet to be explored.

As phytohormones act through a complex network of signal transduction pathways, it is not surprising that mutation of the ubiquitous plant *CK1* altered plant response to multiple relevant phytohormones. Rice *OsCKI1* knockdown mutant displayed abnormal root development with altered auxin content and was also hyposensitive to brassinosteroid (BR) and ABA treatment during germination. Consequently, in *OsCKI1*-deficient plants, the expression level of the genes related to hormone metabolism and signal transduction was altered [31]. Supportively, an investigation of the global phosphorylation dynamics regulated by BRs using mass spectrometry (MS)-based phosphoproteomics revealed that most phosphoproteins had strong connection with BR signaling components, and many substrates were the downstream components of auxin and ABA signaling [66]. Further verification of these proteins would help to elucidate the role of CK1-mediated phosphorylation in BR, which may bridge the downstream events among the phytohormone signaling pathways. 

### 5.4. Plant Stress Response

Due to the sessile lifestyle, plant establishes its stress defense at the level of protection and adaption. Chromatin alterations in chromatin composition and histone modification are emerging as integral and complex elements of stress responses in plants [67,68]. Post-translational modifications of histones including histone phosphorylation appear to be an obvious response of plant chromatin to stress signals. For example, when cultured BY-2 cells of tobacco were exposed to sucrose or NaCl stress, the phosphorylation of histone H3 at S10 and T3 was increased [69], and the dynamic changes of histone were found to be correlated with the upregulation of some stress-inducible genes [25,70,71]. In recent years, CK1 has been found to affect chromatin substructures by phosphorylating histone residue(s). Arabidopsis *mlk1mlk2* double mutant with diminished level of H3T3 phosphorylation was hypersensitive to osmotic stress including salt and drought. *mlk1mlk2* exhibited partial decondensation in pericentromeric/knob heterochromatin, and about one third of *MLK1/MLK2*-dependent genes were stress-related according to the annotation [22]. These findings suggest that changes of MLK-mediated histone phosphorylation occur globally, which provides a direct link between CK1-mediated phosphorylation of histone H3 and stress response. 

In addition to global phosphorylation, CK1 has been reported to be potentially responsible for phosphorylation of the negative regulatory domain (NRD) of DREB2A under normal growth conditions, which in turn facilitates the degradation of DREB2A. Upon heat stress, phosphorylation NRD of DREB2A was decreased, and application of a highly CK1-selective inhibitor caused the accumulation of DREB2A in a dose-dependent manner [50], suggesting that CK1-mediated phosphorylation is likely implicated in response to heat stress by targeting DREB2A. Additionally, Wirthmuelle et al. found that salicylic acid (SA)-induced defense marker genes were strongly upregulated in triple mutant *mlk1, 3, 4,* implying that *MLKs* affect plant immunity via transcriptional regulation of SA signaling [72]. 

Plant *CKL* genes from several species are involved in response to environmental cues, especially extreme temperature. In a high temperature (HT)-sensitive cotton line, for instance, *GhCK1* was induced by HT and overexpression of *GhCK1* resulted in elevated ABA levels [18]. At the early anther development stage, *AtCKL2* and *AtCKL7* were HT-induced due to the binding of AtMYB24 at the promoters [73]. In contrast, a rice dominant allele of *LTG1*, which encodes a CKL protein, conferred low temperature (LT) tolerance, while the recessive *ltg1* allele was hypersensitive to LT [65]. It would be promising to identify the main signaling factors which act as direct targets or functional partners of CK1 in stress response.

## 6. Concluding Remarks

Plants, owing to the sessile nature of their lifestyle, have a large number of CK1 encoding genes including both the canonical *CK1* family members and the *CK1s* unique to the kingdom. The latter ones play fundamental roles in various cellular, physiological and developmental processes by phosphorylating or interacting with target proteins. During the last decade, research has been mainly focused on the regulatory mechanisms of *CK1* in model plants. The findings have considerably advanced our understanding of the genetic regulation network mediated by *CK1*, however, there are still many questions to answer in order to address how plant *CK1* functions. For example, what specifies the plant-specific CK1 from animal CK1? It would be promising to explore the roles of the C-terminus, which is distinct from that of the animals. As some nuclear protein CK1s also reside in the cytoplasm, identification of the non-nuclear targets would be likely to provide insights on CK1 functions in cytoplasm. *CK1s*, especially the *MLK* homologs, are implicated in multiple signaling pathways, but how they orchestrate these pathways is poorly understood. Further investigation of CK1 substrates and interacting partners would help to unveil the biological roles of *CK1* in plant growth and development. 

## Figures and Tables

**Table 1:** The number of Ser/Thr kinases and *MLK* homologs in the angiosperm species.

**Species**		**Genome Size**	**No. of** **Ser/Thr Kinase**	**No. of *MLK* Homolog**
**Eudicotylendons**				
	*Glycine max*	1.1 Gb	29	10
	*Nicotiaa * *attenuata*	2.5 Gb	16	7
	*Medicago * *truncatula*	465 Mb	20	6
	*Populus * *trichocarpa*	500 Mb	17	7
	*Arabidopsis thaliana*	135 Mb	17	4
				
**Monocotylendons**				
	*Triticum * *aestivum*	~17 Gb	21	15
	*Hordeum * *vulgare*	5.3 Gb	8	5
	*Oryza * *sativa*	500 Mb	15	6
	*Sorghum bicolor*	700 Mb	7	7
	*Zea * *mays*	2.4 Gb	8	5
**Lycopodiophyta**				
	*Selaginella * *moellendorffii*	110 Mb	5	4
**Embryophyta**				
	*Marchantia * *polymorpha*	280 Mb	2	1
	*Physcomitrella * *patens*	511 Mb	7	2
**Chlorophyta**				
	*Chlamydomonas * *reinhardtii*	120 Mb	2	2
	*Ostreococcus * *lucimarinus*	13.2 Mb	1	1
**Amborellales**				
	*Amborella * *trichopoda*	870 Mb	3	3

Note: Mut9p protein sequence was used in Blast against the genomes of the indicated species (http://plants.ensembl.org/index.html).

**Table 2:** Summary of the CK1 substrates identified in plants.

CKIs	Substrates	Phosphorylation Sites	Biological Role	Species	References
Mut9p	H3	T3	Repress transcription of euchromatic loci	*Chlamydomonus*	[14]
MLK1/2	H3	T3	Probably for heterochromatic organization maintenance	*Arabidopsis*	[22]
MLK4	H2A	S95	Promote flowering by interacting with CCA1	*Arabidopsis*	[37]
PPK1	CRY2	S506, S523, S525, S526 S598, S599, S605	Destabilize or activate blue-light dependent photoreceptor CRY2	*Arabidopsis*	[16]
PPK1	PIF3	S58, S102, S151-3, S250 S253 S266, S269	Facilitate red light-dependent degradation of photoreceptor PIF3	*Arabidopsis*	[15,54]
PPK1	PIF3	S323, S40/43/45/46, S162, S283-290, S482/T483, T500/T501	Facilitate light-independent degradation of photoreceptor PIF3	*Arabidopsis*	[15,54]
AEL1-4	PYL1	S59, T71, S91, S109, S112, T133S136, T138, S182, S203	Promote ubiquitination and degradation of ABA receptors PYR/PYLs	*Arabidopsis*	[2]
EL1	SLR1	S196, S510	Destabilize SLR1 protein in GA signaling	Rice	[3]
Hd16	Ghd7; PRR37	?	Inhibit photoperiodic flowering	Rice	[4,55]
CK1.3/1.4	CRY2	S587, T603	Promote blue light-induced degradation of CRY2	*Arabidopsis*	[21]
CKL4	PPR5, TOC1	?	Inhibit the expression of PRR5 and TOC1	*Arabidopsis*	[20]
CKL2	ADF4	?	Inhibit actin filament disassembly	*Arabidopsis*	[52]
OsCKL	lipase	?	Regulate lipase activity	Rice	[51,56]
GhCKL	TCP15	?	Regulate *GhPIF4* and disrupts auxin homeostasis	Cotton	[53]
CKL	SebHLH	?	Enhance SebHLH-mediated transactivation of *SeFAD2* gene	Sesame	[17]
CKL	?	?	Function in time keeping	*Ostreococcus*	[7]

Note: MLK1(PPK4/AEL1), MLK2(PPK1/AEL3), MLK3(PPK2/AEL1) and MLK4(PPK3/AEL2) are encoded by At5g18190, At3g03940, At2g25760 and At3g13670, respectively [2,15,22]. ? represents no information available yet.

## Abbreviations

AEL	Arabidopsis *early-flowering* 1-like
CCA1	CIRCADIAN CLOCK-ASSOCIATED 1
CDC7	Cell division control protein 7
CK1	Casein kinase I
CRY2	CRYPTOCHROME 2
DREB2A	Dehydration-responsive element-binding protein 2A
ELF3/4	EARLY FLOWERING 3/4
Ghd7	Grain number, plant height, and heading date 7
H3T3ph	Phosphorylation of histone H3 at threonine 3
Hd16	Heading date 16
LTG1	Low temperature growth 1
MLK	MUT9-like kinase
PIF	PHYTOCHROME INTERACTING FACTOR
PPK	Photoregulatory protein kinase
PRR	PSEUDO-RESPONSE REGULATOR
PYR1/PYL	PYRABACTIN RESISTANCE1/PYR1-LIKE
SLR1	SLENDER RICE 1
TCP15	TEOSINTE BRANCHED1-CYCLOIDEA-PCF transcription factor 15
TOC1	TIMING OF CAB EXPRESSION 1
